# Morphofunctional properties of a differentiated Caco2/HT-29 co-culture as an *in vitro* model of human intestinal epithelium

**DOI:** 10.1042/BSR20171497

**Published:** 2018-04-27

**Authors:** Anita Ferraretto, Michela Bottani, Paola De Luca, Laura Cornaghi, Francesca Arnaboldi, Margherita Maggioni, Amelia Fiorilli, Elena Donetti

**Affiliations:** 1 Dipartimento di Scienze Biomediche per la Salute, Università degli Studi di Milano, Milano 20133, Italy; 2 Centro Ricerca Metabolismi, IRCCS Policlinico San Donato, San Donato Milanese 20097, Italy

**Keywords:** Caco2, Co-culture, Differentiation, HT-29, Transmission electron microscopy

## Abstract

An intestinal 70/30 Caco2/HT-29 co-culture was set up starting from the parental populations of differentiated cells to mimic the human intestinal epithelium. Co-culture was harvested at confluence 0 (T0) and at 3, 6, 10, and 14 days post confluence after plating (T3, T6, T10, and T14, respectively) for morphological and functional analysis. Transmission electron microscopy revealed different features from T0 to T14: microvilli and a complete junctional apparatus from T6, mucus granules from T3, as also confirmed by PAS/Alcian Blue staining. The specific activity of alkaline phosphatase (ALP), aminopeptidase N (APN), and dipeptidyl peptidase IV (DPPIV) progressively increased after T0, indicating the acquirement of a differentiated and digestive phenotype. Transepithelial electrical resistance (TEER), indicative of the barrier properties of the monolayer, increased from T0 up to T6 reaching values very similar to the human small intestine. The apparent permeability coefficient for Lucifer Yellow (LY), along with morphological analysis, reveals a good status of the tight junctions. At T14, HT-29 cells reduced to 18.4% and formed domes, indicative of transepithelial transport of nutrients. This Caco2/HT-29 co-culture could be considered a versatile and suitable *in vitro* model of human intestinal epithelium for the presence of more than one prevalent intestinal cell type, by means of a minimum of 6 to a maximum of 14 post-confluence days obtained without the need of particular inducers of subclones and growth support to reach an intestinal differentiated phenotype.

## Introduction

In order to mimic the human intestinal environment, all assays so far developed employ *in vitro* cell lines, since many experimental difficulties hamper in establishing a long-term primary culture of normal small intestinal and colon cells. Amongst the intestinal cell lines, the ones obtained from tumor region of human colon [1,2], such as HT-29 and Caco2, are the most versatile and used. Both HT-29 and Caco2 cell lines share their origin from colon adenocarcinoma but, when differentiated, they exhibit similar structural and functional features of enterocytes [3–5], but also some relevant differences.

Based on these premises, it is undoubted that one single cell line is not fully representative of the human intestine, neither from a morphological, nor from a permeability point of view. This consideration has driven to develop co-cultures of HT-29/Caco2 cells in order to find an *in vitro* model miming as close as possible the intestinal epithelium. The co-cultures so far proposed in literature were obtained performing two types of methodologies: (i) the use of mucus secreting HT-29 subclones, generally HT29-MTX [6–10]; (ii) the adaptation of these two cell lines to modified growth conditions [11,12]. However, these types of co-culturing present some negative aspects: first, they require time-consuming and long-term growth conditions; second, the cell features and the behavior due to the acquired differentiated phenotype can be hardly distinguished from the ones induced by the medium change.

Therefore, the aim of the present work was to set up a simpler, more versatile but equally useful methodology compared with the ones already published, without the requirement of subclones or exogenous inducers of cell differentiation. The co-culture methodology here proposed is based on the combination of Caco2 and HT-29 parental cells, suitably differentiated according to our established protocols [13,14], in a right proportion, established by preliminary experiments, to obtain a mixed population of enterocytes and mucus secreting cells resembling as far as possible the human intestine. Validity and features of the present co-culture have been studied by morphological analysis to monitor (i) the main ultrastructural structures of differentiated intestinal cells, e.g. microvilli, junctional apparatus, and mucus presence; and (ii) the composition of the intercellular junctions by indirect immunofluorescence. In parallel, we evaluated the alkaline phosphatase (ALP), aminopeptidase N (APN), and dipeptidyl peptidase IV (DPPIV) activity, as known markers of intestinal cell differentiation [5]. The integrity of the tight junctions and the permeability of the cell layer formed were monitored by transepithelial electrical resistance (TEER), together with the apparent permeability of Lucifer Yellow (LY), which is not absorbed by epithelial cells [2,15]. Finally, the exact percentage of the two cell lines during co-culture cell growth and their fates were evaluated through a fluorescent marker.

## Materials and methods

Unless otherwise specified, all cell culture media and reagents were from Sigma–Aldrich (St. Louis, MO, U.S.A.), while FBS was from EuroClone Ltd (West Yorkshire, U.K.).

### Cell cultures

The cell lines HT-29 (BS TCL 132) and Caco2 (BS TCL 87), both from human colon carcinoma, were purchased from Istituto Zooprofilattico Sperimentale di Brescia (Brescia, Italy). HT-29 cells were cultured in 75-cm^2^ plastic flasks (VWR International PBI, Milan, Italy) in Roswell Park Memorial Institute medium 1640 (RPMI 1640) medium supplemented with 10% FBS, 2 mM l-Glutamine, 0.1 mg/l streptomycin, 100.000 U/l penicillin, 0.25 mg/l amphotericin B, containing 13.9 mM glucose. The complete RPMI medium, after cell subcultivation for at least five to six passages, provided a population of differentiated and polarized HT-29 cells [16], with features of absorptive and mucus secreting cells. The differentiation of Caco2 cells was achieved by means of successive subcultivations in Eagle’s minimum essential medium (EMEM) as previously described [14]. Caco2 cells grown in complete RPMI (Caco2 RPMI) were assessed by changing their medium from EMEM to complete RPMI medium after cell plating. Co-culture of Caco2 and HT-29 cells was assessed by plating a mixture of differentiated Caco2 cells from 20th to 40th passage and HT-29 cells from 22nd to 40th passage, in complete RPMI medium to obtain the right percentage. Co-culture was grown for up to 14 days after confluence (T0) and data were obtained for the most of experiments at 3 (T3), 6 (T6), 10 (T10), and 14 (T14) days post confluence, except further specifications. Medium was changed every 3 days from plating.

All cultures were kept at 37°C in 5% CO_2_, 95% air atmosphere and were periodically checked for the presence of mycoplasma. All the experiments were performed with a density of 40000 cells/cm^2^.

### TEM analysis

Cells were seeded in tissue culture dishes (Greiner Bio-One; Cellstar, Frickenhausen, Germany) and maintained in a complete RPMI medium until reaching the correct day of post-confluence. Cells were fixed for 60 min at room temperature (RT) with 3% glutaraldehyde buffered in 0.1 M Sorensen phosphate buffer (pH 7.4). After three washings of 30 min each with the same buffer, cells were post-fixed in 1% osmium tetraoxide (OsO_4_) in 0.1 M Sorensen phosphate buffer, stained with uranyl acetate 2% in H_2_O, progressively dehydrated in 50, 75, 96, 100% ethanol (three passages of 5 min each) and embedded in araldite (Durcupan; Fluka, Milan, Italy), as previously described [13,14]. Ultrathin sections (80-μm thick) were obtained with an Ultracut ultramicrotome (Reichert Ultracut R-Ultramicrotome; Leika, Wien, Austria), stained with lead citrate before examination using a Jeol CX100 electron microscope (Jeol, Tokyo, Japan). On selected electron microphotographs, we measured the length of ten microvilli/picture and results were expressed as mean ± S.D. Statistical significance was set at *P*<0.01.

### PAS/Alcian Blue staining

Cells were seeded in a 24-well plate (BD Falcon Cell Culture Insert PET 1 µm, BD Falcon Companion Tissue Culture Plate, Falcon Corning; Life Science, Durham, U.S.A.) and maintained in complete RPMI medium. Cells were fixed in 4% paraformaldehyde diluted with 0.1 M PBS, pH 7.4 for 20 min at RT, rinsed in PBS, and placed in 70% ethanol. Forceps were used to remove the membranes from the inserts. Membranes were then placed in lens paper, (wrapped up by folding it four times), placed in embedding cassettes and incubated in 70% ethanol for 3 h on a stirrer. Samples were dehydrated through an ascending series of ethanol and paraffin embedding. Sections 4-µm thick were cut with a microtome RM2245 (Leica Microsystems GmbH, Wetzlar, Germany).

Sections were dewaxed and rehydrated through a descending series of ethanol. After rinsing in 3% acetic acid, slides were stained using 1% Alcian Blue pH 2.5 for 15 min, oxidized in 1% periodic acid for 5 min, and rinsed in distilled water. Sections were then immersed in Schiff’s reagent for 5 min, rinsed in 0.5% sodium metabisulphate for 2 min, dehydrated through an ascending series of ethanol, and mounted in Entellan. Images were acquired using a Nikon Eclipse 80i microscope equipped with a digital camera Nikon DS-5Mc (Nikon, Tokyo, Japan) and an image acquisition software (ACT-2U).

### Immunofluorescence analysis of intercellular adhesion

Cells were seeded on glass coverslips, fixed for 5 min at RT with 4% paraformaldehyde in 0.1 mmol/l PBS (pH 7.4), post-fixed in ethanol 70% at 4°C, and stored at –20°C until use. Cells were then washed with PBS (three washes of 5 min each), permeabilized with 0.1% Triton X-100 in PBS for 15 min at RT, and rinsed with PBS at RT. In order to evaluate intercellular adhesion, cells were pre-incubated for 30 min with normal goat serum (1:10) diluted in PBS at RT to saturate the non-specific binding sites and incubated with primary and secondary antibodies as reported in Table 1. Nuclei were counterstained with DAPI (1:50000 dilution in bi-distilled water; 5 min incubation at RT) [17]. For each antibody, a technical negative control was done by omitting primary antibody and replacing it with PBS.

**Table 1:** Summary of the used indirect immunofluorescence protocols

Primary antibody	Dilution/incubation time	Secondary antibody
**E-cadherin (36/E-cadherin BD Bioscience)**	1:500/1 h RT	Goat anti-mouse FITC-conjugated (Jackson Immuno research) 1:200/1 h RT
**Occludin (Invitrogen)**	1:100/overnight 4°C	Goat anti-rabbit Alexa Fluor 488 (Molecular Probes) 1:100/1 h RT
**Desmocollin-2 (Progen)**	1:250/1 h RT	Goat anti-rabbit Alexa Fluor 488 (Molecular Probes) 1:100/1 h RT

### Isolation of cell brush border containing fraction (P2)

Cells were seeded in 75-cm^2^ flasks and, at the indicated time points, cell medium was removed, an ice-cold physiological saline was added and, after shaking, cells were detached with a cell scraper and collected in a centrifuge tube to be finally pelleted after two successive repetitions of the same procedure. Cells extracts were prepared according to a well-described procedure [18,19] with minor modifications. One milliliter of ice-cold Tris/Mannitol buffer (2 mM Tris, Mannitol 50 mM, pH 7.1) was used to homogenize the cell pellets (6–7 mg at T0 and 18–25 mg at T6 of proteins) which were subsequently disrupted by ultrasonication. Before placing the homogenate at 4°C for 10 min and mixed on a rotating plate, CaCl_2_ was added to a final concentration of 20 mM. At the end, samples were centrifuged for the first time (10 min, 950 × ***g***, 4°C) and supernatants were centrifuged again (30 min, 33500 × ***g***, 4°C). The small pellets obtained (fraction P2) contain the brush border membranes and were finally resuspended with a small aliquot of Tris/Mannitol buffer to be used for enzyme activity assays and protein content [20].

### ALP assay, EC 3.1.3.1

The method is based on the *p*-nitrophenol (PNP) production, with color development, from *p*-nitrophenyl phosphate (PNPP), colorless [21].

P2 fractions (2–12 μg) were solubilized with 50 μl Tris/Mannitol buffer; 0.5 ml of the reaction mixture (7 mM PNPP, 0.1 M sodium bicarbonate, and 5 mM MgCl_2_) were added to all the samples. Standard solutions of PNP (0–50 nmoles) were diluted to 0.5 ml with the reaction mixture devoid of PNPP. The reaction was stopped after 25 min at 37°C in the dark, by adding 1 ml of 0.1 M NaOH to each tube. The absorbance was measured at 410 nm and was used to calculate the enzyme activity in reference to the standard curve. Specific activity was expressed as mU/mg protein, one Unit being defined as the enzyme activity that hydrolyses one μmole of substrate per minute. Protein concentration was determined by Lowry method [20].

### DPPIV assay, EC 3.4.14.5

The procedure consists of measuring the absorbance of 2-naphthylamine released from the scission of the substrate Gly-Pro β-naphthylamide [22].

P2 fraction (0.7–4.0 μg) were resuspended to a final reaction volume of 400 μl in 50 mM Tris/HCl buffer, pH 8.4 with 2 mM Gly-Pro β-naphthylamide substrate. Standards solutions of 2-naphthylamine ranging from 0 to 50 μM were prepared using the same buffer. After an incubation period of 30 min at 37°C in the dark, the reaction was stopped by adding 300 μl of 32% trichloroacetic acid (TCA) to each tube. According to Branton–Marshall reaction [23], 100 μl of 0.3% sodium nitrite, 100 μl of 1.5% ammonium sulphamate, and 300 μl of 0.1% N-1-naphthyleyhylenediamine dihydrochloride (in 95% ethanol) were added in sequence to each tube. The absorbance was measured at 560 nm and enzyme activities were calculated according to a standard curve and expressed as mU/mg protein, one Unit being defined as the enzyme activity that hydrolyzes one μmole of substrate per minute. The protein content was measured by means of the Lowry method [20].

### ANP assay, EC 3.4.11.2

The method is based on the chromogenic properties of the 4-Nitroaniline after its separation from l-Ala 4-Nitroanilide substrate [24].

P2 fraction (7–40 μg) were resuspended to a final reaction volume of 1 ml of 30% PBS added with 1 mM CaCl_2_ and 1 mM MgCl_2_ and 1% 10 mM Tris-150 mM NaCl buffer, pH 8 with 1 mM l-Ala 4-Nitroanilide substrate. Standard solutions of 4-Nitroaniline ranging from 0 to 100 μM were prepared using the same buffer. After an incubation period of 30 min at 37°C in the dark, the reaction was stopped at 4°C for 10 min. The absorbance was measured at 405 nm and enzyme activities were calculated according to a standard curve and expressed as mU/mg protein, one Unit being defined as the enzyme activity that hydrolyzes one μmole of substrate per min. The protein content was measured by means of the Lowry method [20].

### TEER

All the cells were seeded and maintained in 24-well plate (Transwell Millicell® Cell Culture Insert PET 1 µm, Millicell® 24-Well Receiver Tray, Millipore Corporation, Billeric, MA, U.S.A.) in their growth medium until the measurement. TEER was measured with a Millicell ERS system (Millipore Corporation) at all the time points indicated above. TEER values in the absence of cells were used as a blank and subtracted from all the cell values. Results were expressed as Ωcm^2^ and represented the average obtained from a minimum of three to a maximum of six wells in which the measurements were done in three distinct regions, then averaged.

### LY permeability study

Co-culture cells were seeded and maintained in 24-well plate (Transwell Millicell® Cell Culture Insert PET 1 µm, Millicell® 24-Well Receiver Tray, Millipore Corporation, Billeric, MA, U.S.A.) in their growth medium.

LY was used to evaluate the apical (A) to basolateral (B) paracellular permeability of Caco2 RPMI and co-culture cells at the sixth day post confluence; 100 µM LY was added at the apical chamber in Hank’s balanced salt solution (HBSS) and incubation for 30, 60, and 120 min was undertaken. At the end of the incubation periods, apical and basolateral solutions were collected and the fluorescence signal was detected by a luminescence spectrometer (PerkinElmer, Beaconsfield, U.K.) at excitation wavelength of 398 nm and emission wavelength of 518 nm. Paracellular permeability, expressed as %LY_basal_ recovery, and the apparent permeability coefficient, *P*_app_, were calculated according to the following equations:

$$\%{\text{LY}}_{\text{basal }}\text{recovery }=\;\frac{{\text{LY}}_{\text{t}}\;(\text{B})}{{\text{LY}}_{0\;}(\text{A})}*100$$

LY_t_ (B) represents the LY concentration in the basolateral chamber (B) at a specific incubation time (t = 30, 60, or 120 min), LY_0_ is the concentration of LY in the apical chamber (A) at the beginning of incubation.

$${\text{P}}_{\text{app}}(\text{cm}/\text{s})=\frac{1}{\text{S}*{\text{C}}_{0}}*\frac{{\text{dQ}}_{\text{t}}}{\text{dt}}$$

S is the surface area of the membrane (0.7 cm^2^), C_0_ is the initial concentration of LY in the apical compartment, Q is the amount of LY molecules transported from the apical to basolateral chamber in a specific period (t = 30, 60, or 120 min).

### Staining of the HT-29 cells in the 70/30 co-culture with days in cultures

Before seeding on glass coverslips placed in a 24-well plate, HT-29 cells were marked with the PKH26 Red Fluorescent Cell Linker Kit for General Cell Membrane Labeling as described by the kit protocol and maintained in their growth medium. This yellow-orange fluorescent dye was stably incorporated in long aliphatic tails into lipid regions of the cell membrane, for a long period [7]. At each time point, the 70/30 co-culture was fixed for 5 min at RT with 4% paraformaldehyde in 0.1 mmol/l PBS (pH 7.4) and washed with PBS (three washes of 5 min each). Nuclei were counterstained with DAPI (1:50000 dilution in bi-distilled water; 5 min of incubation at RT) [17]. The percentage of labeled HT-29 cells compared with total cells was calculated by the following formula:

n° nuclei red cells/n° total nuclei * 100.

### Statistical analysis

**Figure 1:**
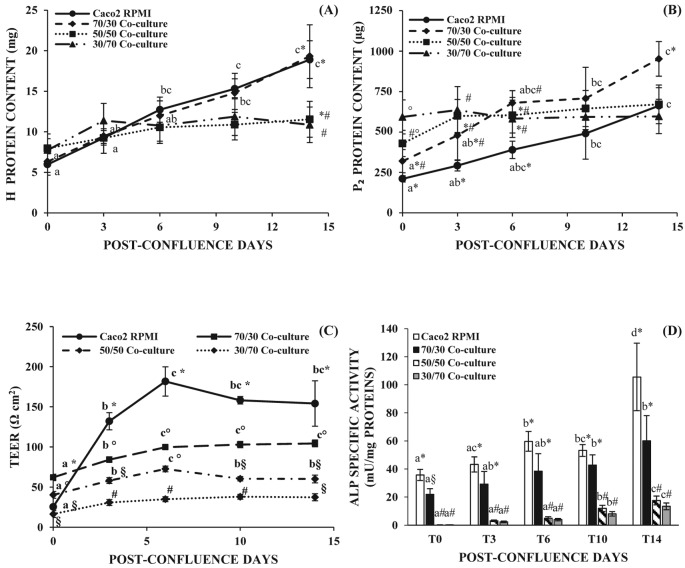
Protein concentration, TEER measurements,and ALP activity of Caco2/HT-29 co-culture at three different seeding cell ratio (**A**) Homogenate (H) and (**B**) P2 protein content determined at different post-confluence days. (**C**) TEER values at different post-confluence days. (**D**) Brush border ALP specific activity at different post-confluence days. Symbols (*, #, §) marked significant differences (*P*<0.01) between different samples. Different letters marked significant differences within the same type of cells but at different time points. (*P*<0.01).

Results shown in Figure 1, 6 and Table 4 represent the mean values and S.D. from at least three independent experiments. Statistically significant differences between mean values were established: (i) by one-way ANOVA followed by a Bonferroni post hoc *t* test with the SPSS 20 statistical software (SPSS, Chicago, IL, U.S.A.) in the case of one cell type at different time points; (ii) by an independent two-sample *t* test in the case of Caco2 RPMI and co-culture cells comparison for each time points. A *P*-value <0.05 was considered significant and was represented by different letters for intrasubject test (within the same type of cells but at different time points) or symbols for intersubject test (between Caco2 RPMI and co-culture cells at the same time point).

## Results

In the first part of the present study, the right proportion of the two cell lines was identified in order to obtain an *in vitro* model of the intestinal epithelium as more similar to the physiological one as possible. The ratios 30/70, 50/50, 70/30 of Caco2/HT-29 cells were chosen based on the different percentage between absorptive enterocytes and goblet cells found along the human intestinal epithelium [25,26] and were therefore analyzed for their main features.

### Cell protein content for the different Caco2/HT-29 co-culture ratio

Protein concentration of the cell homogenate (Η) progressively and similarly increased from T0 to T14 in 70/30 co-culture and Caco2 RPMI, while in the case of 30/70 and 50/50 co-culture a slight increase until T6 and then a plateau was observed (Figure 1A). Protein concentration of P2 fraction interestingly showed a continuous rise from T0 to T14 only in 70/30 co-culture which reached the highest value, while 30/70 and 50/50 co-culture did not show a significant rise in the protein content. Caco2 RPMI cells increased their P2 protein content but this remained always lower than the one assayed for 70/30 co-culture (Figure 1B).

### TEER measurements in the different Caco2/HT-29 co-culture ratio

TEER values of the 30/70 co-culture corresponded to an average value of 35 Ωcm^2^ and did not vary with post-confluence, while the TEER values of the 50/50 co-culture were slightly higher than those observed for 30/70 co-culture (Figure 1C). In the case of 70/30 Caco2/HT-29 co-culture, TEER values rose from T0, reaching a maximum at T6 (*P*<0.01) and continued with an evident plateau without any statistical difference. This trend was observed also for Caco2 RPMI cells but the absolute values of TEER measured, 26–182 Ωcm^2^ for Caco2 RPMI and 62–104 Ωcm^2^ for 70/30 co-culture, were statistically different (*P*<0.01) at all time points after confluence (Figure 1C).

### ALP activity for the different Caco2/HT-29 co-culture ratios

Different brush border associated hydrolase activity can be used to assess the differentiation degree of the cells toward an enterocytic phenotype [5]. In the present study, we have measured the activity of ALP, APN, and DPPIV on the brush border membranes containing P2 fraction. In particular, ALP activity has been assayed to differentiate the different Caco2/HT-29 co-culture ratios since it is considered as an intestinal cell differentiation marker, regardless of the specific cell function.

ALP activity progressively increased with days in culture in both Caco2 RPMI and 70/30 co-culture cells, reaching a statistical significance at T6 for Caco2 RPMI and at T10 for 70/30 co-culture (Figure 1D, *P*<0.05). The absolute values of ALP activity were always higher in Caco2 RPMI than in co-culture, although they reached a statistical significance only at T0 (*P*<0.05). 30/70 and 50/50 co-cultures showed an increase in ALP activity which reaches the significance only at T10 and T14 (*P*<0.01) but the absolute values were always far below the ones obtained in the case of Caco2 RPMI and 70/30 co-culture (Figure 1D).

Results reported in Figure 1, above all the higher ALP activity and TEER determination, have allowed us to individuate the 70/30 Caco2/HT-29 ratio as the most suitable co-culture to develop an *in vitro* model of physiological human intestine. In fact, TEER values of 30/70 and 50/50 co-cultures were indicative of an epithelium too permeable toward the foreign substances, while the TEER values of Caco2 RPMI were indicative of an epithelium too tight toward the physiological absorption. Therefore, the 70/30 co-culture has been deeply studied to confirm our hypothesis.

### TEM analysis for Caco2 RPMI and 70/30 ratio

Ultrastructural observations of parental Caco2 RPMI cells and 70/30 co-culture were summarized in Tables 2 and 3, respectively. Parental Caco2 RPMI (Table 2) formed a continuous monolayer in which cells were less differentiated when compared with Caco2 EMEM [14]. A progressive shift toward a mixed population occurred starting from T3 up to T14.

**Table 2:** Ultrastructural features of parental Caco2 RPMI cells

Time points	Cell junctions	Microvilli	Mucus	Cell arrangement
**T0**	+/−	scattered	−	Continuous monolayer
**T3**	−	scattered	+	Continuous monolayer
**T6**	+	+	+	Continuous monolayer with multilayer
**T10**	+/−	++	++	Continuous monolayer with multilayer
**T14**	++	+/−	++	Multilayer

**Table 3:** Ultrastructural features of 70/30 co-culture cells

Time points	Cell junctions	Microvilli	Mucus	Cell arrangement
**T0**	+/−	+/-	+/−	Continuous monolayer
**T6**	+	+	+	Multilayer
**T14**	++	++	−	Multilayer

**Figure 2:**
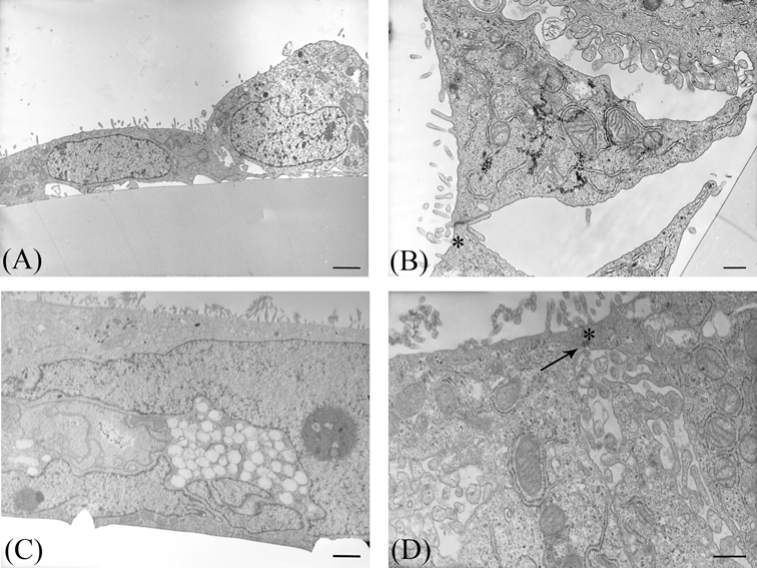
TEM of parental Caco2 RPMI cells Representative photomicrographs of Caco2 RPMI cells araldite ultrathin sections; (**A**,**B**) T0 cells. (**C**,**D**) T14 cells. Arrow indicates a desmosome; asterisks indicate tight junctions. Bars: (A,C) 2 µm; (B,D) 500 nm.

By TEM analysis (TEM), at T0 only scattered microvilli and intercellular junctions were present respectively at the apical and lateral cell surfaces (Figure 2A,B). At T14, a multilayered mixed cell population comprising both absorptive cell type and mucus-secreting cells was clearly evident (Figure 2C). Microvilli, desmosomes, and tight junctions were evident in the uppermost cell layer (Figure 2D).

**Figure 3:**
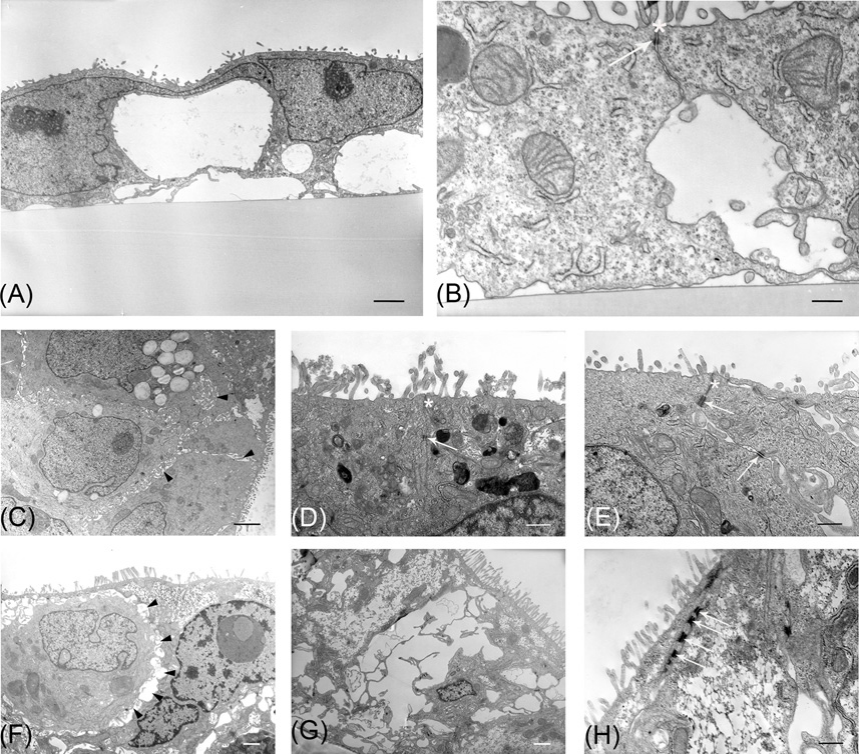
TEM of 70/30 Caco2/HT-29 co-culture at different time points Representative photomicrographs of co-culture araldite ultrathin sections. (**A**,**B**) T0 cells. (**C**–**E**) T6 cells; (**F**–**H**) T14 cells. Arrows indicate desmosomes, asterisks tight junctions, and arrowheads FLS. Bars: (A,C,G) 2 µm; (F) 1 µm; (B,D,E,H) 500 nm.

TEM observations strongly suggested that 70/30 co-culture displayed original features from T0 to T14 (Table 3). At T0, cells were poorly differentiated as indicated by the presence of few and short microvilli (Figure 3A) and scattered intercellular junctions (Figure 3B). Progressively, at T6 a multilayered arrangement was present. Toward the apical surface a cytotype characterized by a well-developed brush border (Figure 3C,D) and a complete junctional apparatus on the lateral membrane was observed (Figure 3D,E). Intercellular spaces in correspondence of the lateral cell membrane, showing small microvilli between adjacent cells (Figure 3C, arrowheads), suggested the presence of follicle-like structures (FLS). In the cell layer, intracellular mucus granules were evident (Figure 3C). At T14, microvilli became more elongated compared with T6 (T6 0.5 ± 0.12 µm compared with T14 1.88 ± 0.34 µm, *P*<0.01) and regular (Figure 3F,G). FLS (Figure 3F, arrowheads) and desmosomes (Figure 3H) were present, while mucus granules were almost absent (Figure 3G).

### PAS/Alcian Blue staining for mucus-secreting cells

The reddish-violet staining indicates the mucus-producing cells and the presence of mucus, while the bluish cytoplasmic background indicates a non-mucus-secreting cytotype.

**Figure 4:**
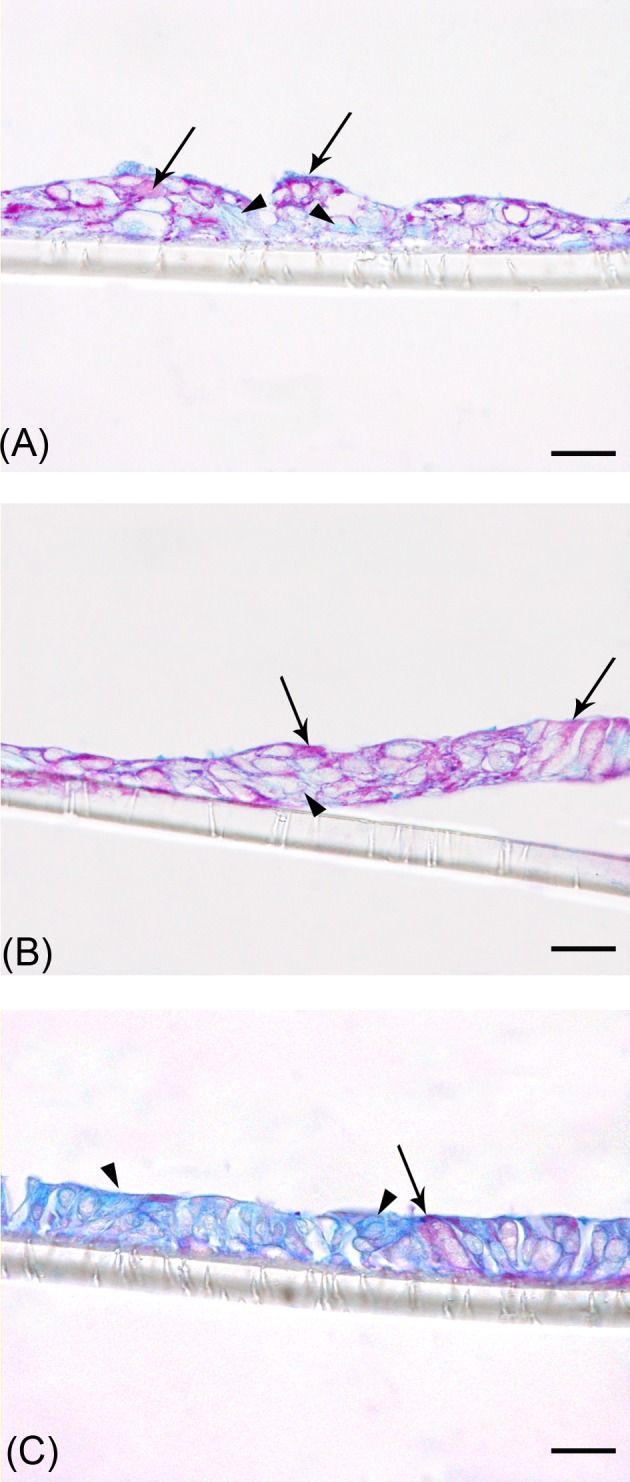
Representative photomicrographs of paraffin sections after PAS/Alcian Blue staining (**A**) Parental Caco2 RPMI cells at T14; (**B**) 70/30 co-culture cells at T6; (**C**) 70/30 co-culture cells at T14. Black arrows indicate cells stained with PAS/Alcian Blue; arrowheads indicate unstained cells. Bars: 10 µm.

Parental RPMI Caco2 cells showed a predominant mucus-secreting phenotype at T14 (Figure 4A), according to TEM results (see Table 2). Interestingly, in the 70/30 co-culture this staining was evident already at T6 (Figure 4B), while at T14, it was almost absent (Figure 4C), in accordance with TEM results (see Table 3). The weak bluish cytoplasmic background indicated that a non-mucus-secreting cell type was always present (Figure 4).

### Immunofluorescence analysis of intercellular adhesion in Caco2 RPMI and 70/30 co-culture

**Figure 5:**
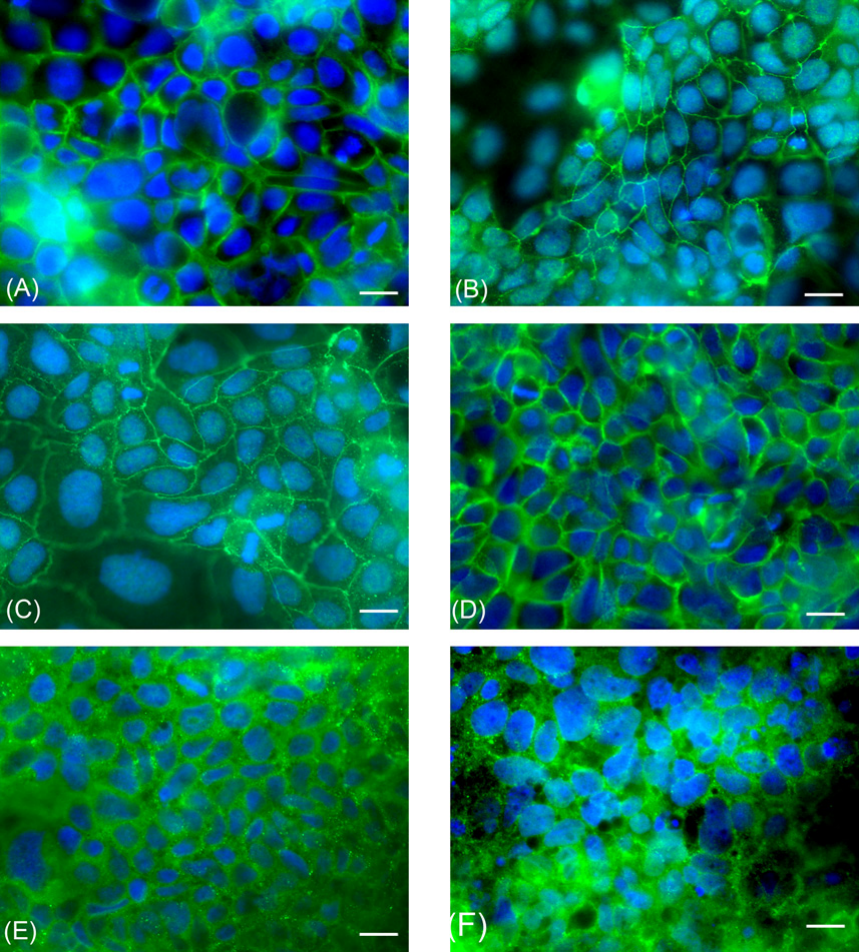
Immunofluorescence analysis of parental Caco2 RPMI cells and 70/30 co-culture Representative photomicrographs of Caco2 RPMI and 70/30 co-culture cells at T6 after intercellular adhesion marker immunostaining. (**A**,**C**,**E**) Caco2 RPMI cells; (**B**,**D**,**F**) co-culture cells. (A,B) occludin. (C,D) E-cadherin. (E,F) Dsc-2. Bars: 10 µm.

By immunofluorescence, we evaluated the expression of occludin, E-cadherin, and Desmocollin-2 (Dsc-2) transmembrane proteins in Caco2 RPMI and 70/30 co-culture cells at the different time points to assess the presence of tight junctions, adherens junctions, and desmosomes, respectively (Figure 5). In all experiments, immunoreactivity was always localized in correspondence of the cell membrane. At all considered time points, immunofluorescence analysis of Caco2 RPMI and 70/30 co-cultures always indicated the presence of tight, adherens junctions, and desmosomes as demonstrated by occludin, E-cadherin and Dsc-2 immunopositivity.

### APN and DPPIV activity in 70/30 co-culture

**Figure 6:**
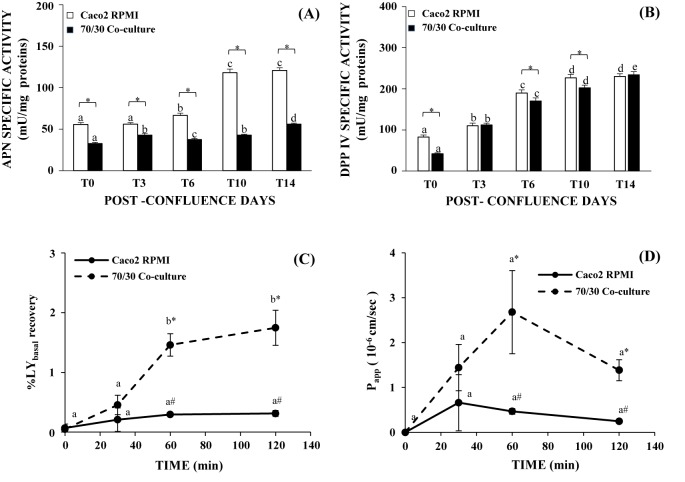
ANP, DPPIV specific activity and permeability study for 70/30 co-culture (**A**) Brush border ANP and DPPIV, (**B**) specific activity of Caco2 RPMI and 70/30 co-culture at different post-confluence days. Different letters marked significant differences within the same type of cells but at different time points (*P*<0.05). Asterisk indicates statistically significant difference between the two cell cultures (between Caco2 RPMI and co-culture cells at the same time point, *P*<0.05). (**C**) %LY recovery in the basolateral chamber of Caco2 RPMI and 70/30 co-culture at T6 after different incubation times (0, 30, 60, and 120 min). (**D**) Caco2 RPMI and 70/30 co-culture at T6 *P*_app_ values after different incubation times (0, 30, 60, and 120 min). Different letters marked significant differences within the same type of cells but at different time points (*P*<0.01) (A); while symbols indicates statistically significant difference (between Caco2 RPMI and co-culture cells at the same incubation time, *P*<0.01 (A); *P*<0.05 (B).

APN activity in Caco2 RPMI increased starting from T6 and reaching a maximum at T10 (Figure 6A, *P*<0.05), while in 70/30 co-culture cells the APN activity was slightly different from T0 up to T14 when it became definitely higher than the other time points (*P*<0.01). The APN activity values were always significantly higher in Caco2 RPMI than in 70/30 co-culture cells (*P*<0.01). DPPIV activity increased from T0 to T10 in Caco2 RPMI and from T0 to T14 in co-culture cells (Figure 6B, *P*<0.05), with absolute values very similar between the two cell population and statistically different (*P*<0.05) only at T0, T6, and T10.

### LY permeability for 70/30 co-culture

Intestinal permeability is a key factor when an *in vitro* intestinal model has to be used for drug absorption and/or cytotoxicity studies. From our data about mucus and junctional apparatus presence, it can be supposed that 70/30 co-culture at T6 could display the best properties. To confirm this hypothesis, we further investigated the permeability features by studying the passage of a drug, i.e. LY.

An increase in LY concentration was assayed in the basolateral chamber of 70/30 co-culture at T6, more pronounced in the first 60 min, as evidenced by the statistical significance between 30 compared with 60 min (*P*<0.01) (Figure 6C). On the contrary, the percentage of LY recovery in the basolateral compartment of Caco2 RPMI remained in the same range for all the incubation times. Statistically significant differences (*P*<0.01) were present after 60 and 120 min of LY incubation between the 70/30 co-culture and Caco2 RPMI. *P*_app_ evaluation ranged from 0.2 × 10^−6^ to 0.7 × 10^−6^ cm/s for Caco2 RPMI and 1.4–2.7 × 10^−6^ cm/s for co-culture without changes amongst the different incubation times. Results revealed statistical significant differences (*P*<0.05) between 70/30 co-culture and Caco2 RPMI after 60 and 120 min of incubation (Figure 6D).

### Identification of HT-29 cell proportion and fate in the 70/30 co-culture in correlation with post-confluence days

**Figure 7:**
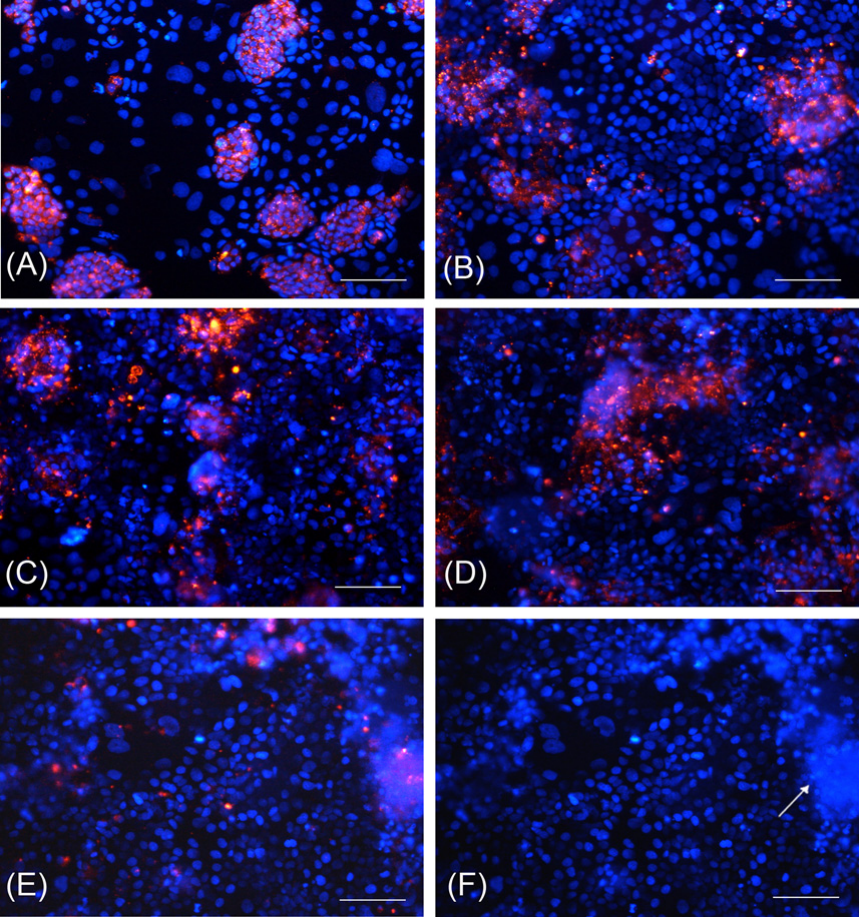
HT-29 cell staining and nuclei identification in 70/30 co-culture at different post-confluence days (**A**) T0 cells; (**B**) T3 cells; (**C**) T6 cells; (**D**) T10 cells; (**E**) T14 cells; (**F**) domes formed by HT-29 cells at T14. Red fluorescence: HT-29 cells marked with PKH26 GL; blue fluorescence: nuclei counterstained with DAPI. White arrow indicates domes. Bars: 100 µm.

At T0, 59.3 ± 4.5% of total cells were identified as HT-29 cells. This percentage decreased to 30.4 ± 4.8 at T3 (*P*<0.01) to finally reached the lower value (18.4 ± 6.4) at T14 (Table 4 and Figure 7). At T14, the appearance of domes formed by HT-29 cells was also showed (Figure 7F).

**Table 4:** HT-29 and Caco2 percentage in the 70/30 co-culture with days in culture

Time points	% HT-29	% Caco2
**T0**	59.3 ± 4.5	40.7 ± 4.5
**T3**	30.4 ± 4.8*	69.6 ± 4.8*
**T6**	22.2 ± 5.2*	77.8 ± 5.2*
**T10**	27.1 ± 10.2*	72.9 ± 10.2*
**T14**	18.4 ± 6.4*	81.6 ± 6.4*

Mean values ± S.D. coming from ten images for each time point and calculated as reported in ‘Staining of the HT-29 cells in the 70/30 co-culture with days in cultures’ section. Asterisk marked any significant difference with respect to T0 (*P*<0.01).

## Discussion

The present paper reports the standardization of an original experimental setting of co-culturing Caco2 and HT-29 cells characterized by a simple and fast methodology reaching morphofunctional features similar to the *in vivo* human small intestinal epithelium, without employing the specific HT29-MTX subclone to obtain mucus-secreting cells [8,9,12,25], a 3D culture [27], particular growth factors, and supports.

The necessity of a co-culture comes from the impossibility to represent, as better as possible, with an *in vitro* cell model the human intestinal epithelium using a single cell line. HT-29 cells are considered a population with a high degree of heterogeneity due to the formation of absorptive and goblet cells mixture independent of the growth conditions [5,13]. The permeability of tight junctions in mucus secreting HT-29 subclones reaches values different from the normal intestine [7,8]. On the contrary, Caco2 cells grown in standard medium display clear features of an intestinal differentiation toward an absorptive phenotype devoid of mucus granules [4,14]. Nonetheless, the permeability of Caco2 monolayer is lower than the physiological human intestine.

The percentage of the two main intestinal cell types, absorptive and mucus secreting, changes in the different gut regions since at the level of duodenum, the epithelium is formed by 90% absorptive cells and 10% goblet cells, while in large intestine goblet cells reach the 24% [25,26]. On this basis, the ratio 30/70, 50/50, and 70/30 Caco2/HT-29 cells have been initially considered since: (i) whole populations of Caco2, specifically enterocytes, and HT-29, characterized by a high degree of cell heterogeneity, were used; (ii) the two cell type proportions can rapidly modify from seeding time since it also depends on exogenous factors, such as adherence to the growth supports, proliferation rate, and changing of the original phenotype of the cells with days in culture.

The 70/30 Caco2/HT-29 seeding ratio of parental cells in a co-culture proved to be the most suitable since it was found to have the highest protein content in the cellular subfraction containing microvilli and membranes above all at T14, when TEM revealed a marked presence of microvilli. Moreover, ALP activity, indicative of the differentiated degree of intestinal cells, was higher than the 30/70 and 50/50 cell ratio. In addition to these data, TEER measurements, an indirect index of the cell permeability, showed absolute values in the range of small human intestine [28], while both Caco2 RPMI and 30/70–50/50 cell ratios showed different values. The importance of the presence of HT-29 cells is also underlined by their proportion and fate in the 70/30 co-culture along the post-confluence days. Although the seeding percentage of the two cell lines was 70/30, at T0 HT-29 cells are the most abundant cells, due to their higher proliferation rate, but from T3 on they approximately reached the 70/30 ratio. At T14 HT-29 cells reduced their proportion to only the 18.4% and at the same time two morphological signs appeared: the mucus disappearance and the domes of HT-29 cells. The last structures are considered very specialized cell organization in charge of transepithelial transport properties of nutrients [5]. Based on these results, we can speculate that the HT-29 cells still present at T14 shift their functional features toward the absorptive phenotype.

The reduction in the post-confluence days needed to obtain a well-differentiated intestinal cell model compared with the previously reported studies is an interesting feature of the present 70/30 co-culture and could be explained by the combination of two Caco2 cell differentiation pathways: (i) the post-confluence [4] and (ii) the number of passages higher than 20 [14].

The combination of two different forces mainly drove the fast and marked differentiation of our 70/30 co-culture: the presence of HT-29 cells and the use of RPMI medium. HT-29 cells exerted a key role by promoting a well-developed co-culture phenotype under RPMI subcultivation, a condition necessary to induce the switch of undifferentiated to mucus-secreting cells. At the same time, the cell growth condition in RPMI *per se* was not enough to differentiate Caco2 cells, since under this culture condition they seemed more refractory to differentiate than both co-culture and Caco2 cells cultured in EMEM, as previously described [14].

The morphological data here reported evidenced two major time points: T6 and T14. The coexistence of a differentiated absorptive phenotype and mucus-secreting cells since T6 represents the major advantage of the present 70/30 co-culture respect to the previously reported data [4]. The presence of mucus, at this post-confluence day, is determinant not only for a correct barrier function, but also for the permeability of the molecules derived from food digestion and present in the intestinal lumen [29,30]. At T14, the absence of mucus, the well-developed brush border and the complete junctional apparatus stand for a well-differentiated epithelium with a prevalent absorptive phenotype. The formation of a multilayer at this time point was in agreement with most of the reported methods set up to differentiate intestinal cells, from the origin [4] to the most recent ones [11,12]. As it concerns the presence of FLS in our co-culture, the FLS displayed a brush border with functional microvilli and the ability to transport ions and water toward the lumen. Cells involved in the FLS formation display a differentiated phenotype characterized by absorption, secretion, and excretion functions. These data are in agreement with the observations of Mirrione et al. [31] describing the FLS, for the first time in an intestinal cell line, as a large inter- or intracellular structure, which appears spontaneously in polarized cells with a complete junctional apparatus.

Also, the permeability degree of 70/30 co-culture is indicative of differentiated intestinal cells. In fact and noteworthy, in our experimental conditions, the TEER absolute values of 70/30 co-culture were more similar to those of the human small intestine, i.e. 50–100 Ωcm^2^ (duodenum, jejunum, and ileum) as previously reported [28]. LY can pass through cell junctions and is used to evaluate drug passive absorption giving direct assessment of the permeability displayed by our 70/30 co-culture. The LY *P*_app_ values suggested the presence and a good status of the tight junction apparatus [32,33]. These values were higher than those showed by Caco2 RPMI cells, in accordance with literature [12]. Despite the diversity of TEER values obtained for Caco2 RPMI and 70/30 co-culture at T6, there were small differences in the paracellular transport of LY, in accordance with previous studies showing that a large change in TEER values is associated with no or little change in the paracellular transport of different molecules, as TEER is mainly an indicator of ion passage through tight junctions [32,33].

The suitability of the present co-culture as an *in vitro* intestinal model is based on the strict correlation between the morphological and biochemical data presented here, although it has been described as a very difficult process to obtain in intestinal cells [34]. The first morphofunctional correlation supporting differentiation and suitability of our 70/30 co-culture was the progressive and parallel increase from T0 to T14 of the microvilli and the brush border associated hydrolase activity. At long-term culture condition (T14) co-culture exhibited a well-differentiated epithelium with a prevalent absorptive phenotype due to the well-developed microvilli and absence of mucus. These morphological features were in correlation with a progressive switch toward an enterocytic phenotype supported by a parallel increase in the ALP, APN, and DPPIV activity. While ALP activity can be detected also in colonocytes, although less than in enterocytes [35], DPPIV is not expressed in human adult colon [36], thus the increasing activity from T0 up to T14 is very significant.

The second morphofunctional correlation was between the junctional apparatus development and TEER. During cell differentiation, the decrease in permeability is considered a marker of tight junction maturation. At T6, 70/30 co-culture displayed abundant tight junctions and mucus, morphological features associated with the maximum TEER value, therefore to the minimum value of cell permeability. The progressive decrease in TEER from T6 was supported by the increasing presence of desmosomes, which do not contribute to electrical resistance development as tight junctions do.

The present work could be considered as a follow-up of our previous standardized cell differentiation protocols, leading to obtain a co-culture in which morphological and biochemical features of co-cultured parental cells changed with time, strongly suggesting a progressive and active interaction, a condition more favorable compared with the co-cultures in which the considered microenvironment is stationary.

The real applicability of the present co-culture model has just been demonstrated in three separate studies in which the 70/30 co-culture at T6 was employed to determine the possible cytotoxicity and the digestion products of some foods [37–40].

## Conclusion

For each time point considered here, the Caco2/HT-29 70/30 co-culture revealed a specific degree of cell differentiation, permeability properties and protease activity, thus offering the possibility of selecting a specific time point depending on the experimental aim. In fact, the minimum value of cell permeability together with tight junctions and mucus presence indicate T6 as a suitable post-confluence time for studies about drug and/or food permeability, whereas the increase in the number of microvilli together with the major protease activity indicate T14 as a good model for digestion study.

In conclusion, the 70/30 co-culture setting here presented revealed useful morphofunctional features of *in vitro* model of human intestinal epithelium suitable for drug transport and nutrient absorption studies, especially when a possible alteration of cell function or cell permeability occurs.

## Abbreviations

ALP: alkaline phosphatase
APN: aminopeptidase N
Caco2 RPMI: Caco2 cells grown in complete RPMI
DPPIV: dipeptidyl peptidase IV
Dsc-2: desmocollin-2
EMEM: Eagle’s minimum essential medium
FLS: follicle-like structure
LY: Lucifer yellow
PNP: *p*-nitrophenol
PNPP: *p*-nitrophenyl phosphate
RPMI 1640: Roswell Park Memorial Institute medium 1640
RT: room temperature
TEM: TEM analysis
T0: day of the attainment of 100% confluence
TEER: transepithelial electrical resistance
